# The source of SYBR green master mix determines outcome of nucleic acid amplification reactions

**DOI:** 10.1186/s13104-016-2093-4

**Published:** 2016-06-04

**Authors:** Jianxin Yang, Berit Kemps-Mols, Marijke Spruyt-Gerritse, Jacqueline Anholts, Frans Claas, Michael Eikmans

**Affiliations:** Department of Immunohematology and Blood Transfusion, Leiden University Medical Center, Bldg 1, E3-Q, Albinusdreef 2, 2333 Leiden, The Netherlands

**Keywords:** Quantitative PCR, Specificity, Genomic DNA, mRNA, High resolution melting, Performance

## Abstract

**Background:**

Quantitative (q) PCR by amplification of nucleic acid with a fluorescent dye is widely used. Selection of adequate PCR reagents and devices is relevant to achieve reliable and consistent data. Our main objective was to test the robustness of different commercial SYBR green PCR mixes with respect to specificity and sensitivity of the PCR assay, across various PCR machines (Light Cycler 96, ViiA7) and amplification protocols. Herein, we applied PCR protocols for determining mRNA transcript levels, DNA copy numbers, and DNA genotype.

**Results:**

First, we set up 70 primer-based assays that targeted immune-related mRNA transcripts. Of the 70 assays 66 (94.3 %) resulted in a single melting curve peak, indicating specificity of the amplification, with PCR mixes from large vendors (Roche, ABI, Bio-Rad). But this was only seen when the PCR protocol that was indicated in the vendor’s guidelines for each particular mix was applied. When deviating from the prescribed protocol, suboptimal melting curves were most often seen when using Roche SYBR green. With respect to PCR yields, the use of ABI mix more often led to lower Cq values. Second, we set up 20 primer-selective PCR assays to target different insertion-deletion and single nucleotide polymorphism regions throughout the genome. The variation in delta Cq between positive and negative DNA samples among the PCR assays was the lowest when using ABI master mix. Finally, the quality of high resolution melting (HRM) assays for DNA genotyping was compared between four commercial HRM PCR mixes (Roche, Bioline, PCR Biosystems, ABI). Only Roche and ABI mixes produced optimal clusters of melting profiles that clearly distinguished genotype variants.

**Conclusions:**

The current results show a preference for the use of ABI mix when it comes to obtaining higher sensitivity in cDNA analysis and a higher consistency among assays in distinguishing DNA genotypes among different individuals. For HRM assays, it is advisable to use master mix from a relatively large vendor.

**Electronic supplementary material:**

The online version of this article (doi:10.1186/s13104-016-2093-4) contains supplementary material, which is available to authorized users.

## Background

Real-time polymerase chain reaction (PCR) is widely used to measure gene expression and DNA copies [1, 2]. The most commonly used methods for quantitative polymerase chain reaction (qPCR) are based on non-specific SYBR green chemistry and specific Taqman probe chemistry [3]. Intercalating dyes, which bind double-stranded (ds) DNA with high efficiency in the reaction, are most commonly used. When it binds to dsDNA, the fluorescence signal enhances >1000-fold compared to situation where it is unbound and in free solution [4, 5]. The overall fluorescence intensity is proportional to the amplified products and increases as the target is amplified [6]. A drawback of SYBR green I is its lack of specificity: binding to nonspecific dsDNA in the real-time PCR reaction hampers reliable quantification of the specific product [7]. Presence of non-specific PCR products can be ruled out by performing a melting curve analysis [8]. Therefore, the use of DNA-binding dyes may require more extensive optimization. In general, when performing singleplex assays the use of SYBR green dye is preferable over that of probe chemistry, since the former assays are easier to design, faster to set up, and less expensive [9, 10].

High resolution melting (HRM) analysis is a novel, closed-tube, high-throughput technology for identifying mutations and polymorphisms in nucleic acid sequences [11, 12]. The combination of a saturating, DNA-binding dye with superior instrumentation and sophisticated software enables the detection of genetic variations by analyzing PCR melting curves at a finer temperature resolution [11]. HRM reactions generate specific and sensitive melting profiles. They can be used for genotyping, mutation screening, and methylation analysis based on heterozygosity, length, and GC content [13].

Numerous real-time PCR devices and master mixes are available on the market. To perform reliable high-quality data, PCR master mix, and equipment need to be optimal. However, general lab optimized protocols are widely used for different gene targets and performed diversely between conditions. Our main objective was to test the robustness of different commercial SYBR green PCR mixes with respect to specificity and sensitivity of the PCR assay. This was tested across various PCR machines and amplification protocols for assessment of mRNA transcript levels, DNA copy numbers, and DNA genotypes.

## Methods

### PCR machines, SYBR green mixes and HRM mixes

Equipment used included the Light Cycler 96 (Roche Diagnostics, Mannheim, Germany) and the ViiA 7 (Applied Biosystems by Life Technologies, Austin, TX, USA) real-time PCR machines. Performance of three different PCR mixes was compared, including SYBR Select Master Mix (Applied Biosystems), iQ SYBR green supermix (Bio-Rad, Hercules, CA, USA), and FastStart essential DNA Green Master (Roche Diagnostics). We evaluated four different HRM mixes on the lighter cycler 96, namely high resolution melting master (Roche Diagnostics), SensiFast HRM Kit (Bioline, London, UK), qPCRBIO HRM Mix(PCR Biosystems, London, UK), and MeltDoctor HRM Master Mix (Applied Biosystems).

### Nucleic acid extraction and cDNA synthesis

DNA was isolated using chemagic DNA Blood2k Kit by chemagic MSM I equipment (PerkinElmer), and the quantity was measured on a NanoDrop 2000 Spectrophotometer (Thermo Fisher Scientific Inc, Asheville NC). Isolated DNA samples were diluted to 10 ng/µl with nuclease-free water and used as template in qPCR and HRM assays.

RNA was extracted using the NucleoSpin miRNA kit (Macherey–Nagel, Germany) from peripheral blood cells obtained by ficoll or percoll gradients, namely cell subsets positive for either CD3 (T cells) or CD14 (monocytes). Protocols for total RNA purification were followed as described by the manufacturer. RNA quantity was determined on a NanoDrop 2000 Spectrophotometer. RNA quality was evaluated using the StdSense Analysis kit and the Experion RNA analyzer (Bio-Rad, Hercules, CA). Complementary DNA was synthesized from 150 ng of total RNA (RNA quality index >7.0) following the manufacturer’s manuals: Superscript III RT (Invitrogen; 200 U of RT), 0.5 mM dNTP, 40 U of RNAse OUT, and 5 mM DTT. RNA was combined with oligo-dT (Invitrogen; 0.25 mg) and random nucleotide hexamers (Invitrogen; 0.25 mg), and incubated at 65 °C for 5 min [14]. The tubes were immediately placed on ice after incubation, and the remaining constituents were added. The reactions were allowed to proceed at 25 °C for 5 min, at 50 °C for 60 min, and then terminated at 70 °C for 5 min.

### PCR primers

Optimal primers pairs for cDNA assays were selected using Primer 3 version 4.0.0 [15, 16] or Universal Probe Library. To prevent amplification of genomic DNA, forward and reverse primers for majority of the transcripts were designed to target separate exons, spanning at least one intron with a size of 800 bp or more. The PCR efficiency of amplification was calculated by the software using the four-fold serial dilution of pooled cDNA, and 90–110 % was considered as acceptable. The primer selection for genomic DNA (gDNA) assays (S01a, S01b, S03, S04a, S04b, S05a, S05b, S06, S07a, S07b, S08a, S08b, S09a, S09b, S10a, S10b, S11a) was based on a previous study (Table 1) [17]. Firstly, high percentage of heterozygous biallelic polymorphism in the general population was selected. Second, one of the primer sequences was specific to each allele of polymorphic site, whereas the other one was picked in a common region. HRM primers were designed to amplify a short DNA segment covering polymorphism rs2230199.

**Table 1:** Primer sequences and amplification efficiency

Target	Forward primer (5′–3′)	Reverse primer (5′–3′)	Amplicon (bp)	Reagent	Efficiency
GAPDH	ACCCACTCCTCCACCTTTGAC	TCCACCACCCTGTTGCTGTAG	110	ABI	0.98
TLR2	GTGATAGGTGTGAGGCAGGT	GTGGCCGCCTTGATTCATAG	136	ABI	0.93
CD1c	TTTCTGCAGTTTCTGCTGCTA	GAGACGTGTTCCTGGGATG	74	ABI	1.06
CD54	CCTTCCTCACCGTGTACTGG	AGCGTAGGGTAAGGTTCTTGC	90	ABI	1.05
CD68	TTCCCCTATGGACACCTCAG	TTGTACTCCACCGCCATGTA	86	ABI	1
CCL4	CCTGCTGCTTTTCTTACAC	CACAGACTTGCTTGCTTC	126	ABI	1.09
IL4	GTCTCACCTCCCAACTGCTT	GTTACGGTCAACTCGGTGCA	157	Bio rad	0.99
IL4	GTCTCACCTCCCAACTGCTT	GTTACGGTCAACTCGGTGCA	157	Roche	1.01
IL8	GAAGGAACCATCTCACTG	CCACTCTCAATCACTCTC	200	Bio rad	0.96
IL8	GAAGGAACCATCTCACTG	CCACTCTCAATCACTCTC	200	Roche	0.94
IL1RN	CCTGTCCTGTGTCAAGTCTGG	AGCGGATGAAGGCGAAGC	110	ABI	0.93
CEBPB	CGCTTACCTCGGCTACCA	ACGAGGAGGACGTGGAGAG	65	ABI	0.94
IL-18	TGCATCAACTTTGTGGCAAT	ATAGAGGCCGATTTCCTTGG	169	ABI	1
V-FOS	ACTACCACTCACCCGCAGAC	CCAGGTCCGTGCAGAAGT	75	ABI	0.98
Egr-1	AGCCCTACGAGCACCTGAC	GGTTTGGCTGGGGTAACTG	92	ABI	0.9
Egr-2	TTGACCAGATGAACGGAGTG	TGGTTTCTAGGTGCAGAGACG	121	ABI	0.92
CD43	AAGATGTCATCAGTGCCCCA	CACGGTGTGGGATCCTAGAG	90	ABI	0.93
CCR7	GGTGGTGGCTCTCCTTGTC	ACTGTGGTGTTGTCTCCGATG	84	ABI	1.1
CD40	GCAGGCACAAACAAGACTGA	ATGGCAAACAGGATCCCGAA	95	ABI	0.91
S01a	GGTACCGGGTCTCCACATGA	GGGAAAGTCACTCACCCAAGG			
S01b	GTACCGGGTCTCCACCAGG	GGGAAAGTCACTCACCCAAGG			
S03	CTTTTGCTTTCTGTTTCTTAAGGGC	TCAATCTTTGGGCAGGTTGAA			
S04a	CTGGTGCCCACAGTTACGCT	AAGGATGCGTGACTGCTATGG			
S04b	CTGGTGCCCACAGTTACGCT	AGGATGCGTGACTGCTCCTC			
S05a	AAAGTAGACACGGCCAGACTTAGG	CATCCCCACATACGGAAAAGA			
S05b	AGTTAAAGTAGACACGGCCTCCC	CATCCCCACATACGGAAAAGA			
S06	CAGTCACCCCGTGAAGTCCT	TTTCCCCCATCTGCCTATTG			
S07a	TGGTATTGGCTTTAAAATACTGGG	TGTACCCAAAACTCAGCTGCA			
S07b	GGTATTGGCTTTAAAATACTCAACC	CAGCTGCAACAGTTATCAACGTT			
S08a	CTGGATGCCTCACTGATCCA	TGGGAAGGATGCATATGATCTG			
S08b	GCTGGATGCCTCACTGATGTT	TGGGAAGGATGCATATGATCTG			
S09a	GGGCACCCGTGTGAGTTTT	TCAGCTTGTCTGCTTTCTGGAA			
S09b	GGGCACCCGTGTGAGTTTT	CAGCTTGTCTGCTTTCTGCTG			
S10a	GCCACAAGAGACTCAG	TGGCTTCCTTGAGGTGGAAT			
S10b	TTAGAGCCACAAGAGACAACCAG	TGGCTTCCTTGAGGTGGAAT			
S11a	TAGGATTCAACCCTGGAAGC	CCAGCATGCACCTGACTAACA			
Hy	TTCTGGAACCTTTCTTTTCAGGC	ACTTCCCTCTGACATTACCTGATAATTG			
HA-8p	TGCAGTCAGCAGATCACCC	CTTCTGGGCAACAGTTATGGA			
KIR3 DS1	CATCRgTTCCATgATgCg	CCACgATgTCCAggggA			
	TCCATCggTCCCATgATgTT				

### qPCR and HRM assays and PCR protocols

The 20-µL qPCR reaction system (cDNA assays) contained 4 µL of 25-times-diluted cDNA, 10 pmol forward and reverse primers, 10 µL of PCR Mix, and nuclease-free water. The 20-µL qPCR reaction (DNA assays) included 50–200 ng DNA, 10 µL of SYBR PCR Mix, 6 pmol forward and reverse primers, and nuclease-free water. The Roche HRM master mix reaction consisted of 7.5 µl of mix, 3 pmol forward and reverse primers, 3 mM MgCl2, 20 ng DNA, and nuclease-free water. Besides, the 15-µl HRM PCR reaction consisted of 7.5 µl of HRM mix, 6 pmol forward and reverses primers, 20 ng DNA, and nuclease-free water.

The PCR program (cDNA assays) strictly followed the prescribed protocols for each PCR mix (Table 2). Upon completion of each run, a melting curve analysis was performed to check specificity of the primers. In some occasions, the PCR product was additionally analyzed by agarose gel electrophoresis. The quantification cycle (Cq) value represents the number of cycles needed to reach a set threshold fluorescence signal level, which is a measure of number of cDNA or DNA copies.

**Table 2:** Prescribed PCR amplification program

Mix	Steps	Temperature °C	Duration	Cycles
ABI	UDG activation	50	2 min	Hold
	Activation	95	2 min	Hold
	Denature	95	15 s	40
	Anneal/extend	60	60 s	
	Melt curve analysis	95	10 s	
		60	60 s	
		97	5 s	
BioRad	Activation	95	3 min	Hold
	Denature	95	15 s	40
	Anneal/extend	60	45 s	
	Melt curve analysis	95	10 s	
		55	60 s	
		95	15 s	
Roche	Activation	95	10 min	Hold
	Denature	95	10 s	40
	Anneal	60	10 s	
	Extend	72	10 s	
	Melt curve analysis	95	10 s	
		65	60 s	
		95	15 s	
General lab PCR program	Activation	95	10 min	Hold
	Denature	95	15 s	45
	Anneal/extend	60	60 s	
	Melt curve analysis	95	10 s	
		55	60 s	
		97	5 s	

The ramp of each machine were set to default

The HRM PCR program consisted of a pre-incubation for 10 min, followed by 45 cycles of denaturation at 95 °C for 10 s, annealing at 60 °C for 15 s, and extension at 72 °C for 15 s. Melting analysis was performed by first heating to 95 °C for 1 min, cooling to 40 °C for 1 min, heating to 65 °C, and then melting with continuous acquisition (15 readings/°C) of fluorescence signal until 97 °C. Fifteen DNA samples were analyzed, 12 of which were homozygous (GG) and three of which were heterozygous (GC) at the SNP location.

### Availability of data and materials

The datasets supporting the conclusions of this article are available in the figshare repository 10.6084/m9.figshare.3207802 and https://www.figshare.com/articles/The_source_of_SYBR_Green_master_mix_determines_outcome_of_nucleic_acid_amplification_reactions/3207802. The dataset supporting the conclusions of this article is included within the article (and its Additional file 1).

### Ethics (and consent to participate)

Written informed consent was obtained from donors for use of part of the human material for scientific purposes. Samples were processed and analyzed in an anonymous way. Blood samples used for nucleic acid analysis were obtained in the context of studies performed in accordance with the Declaration of Helsinki Good Clinical Guidelines and approved by the local medical ethics committee.

### Data analysis

Statistical analyses were performed using SPSS statistics 20. The mean delta Cq values (positive minus negative gDNA samples) between PCR mixes were compared by paired T test.

## Results

### Amplification of cDNA

**Fig. 1:**
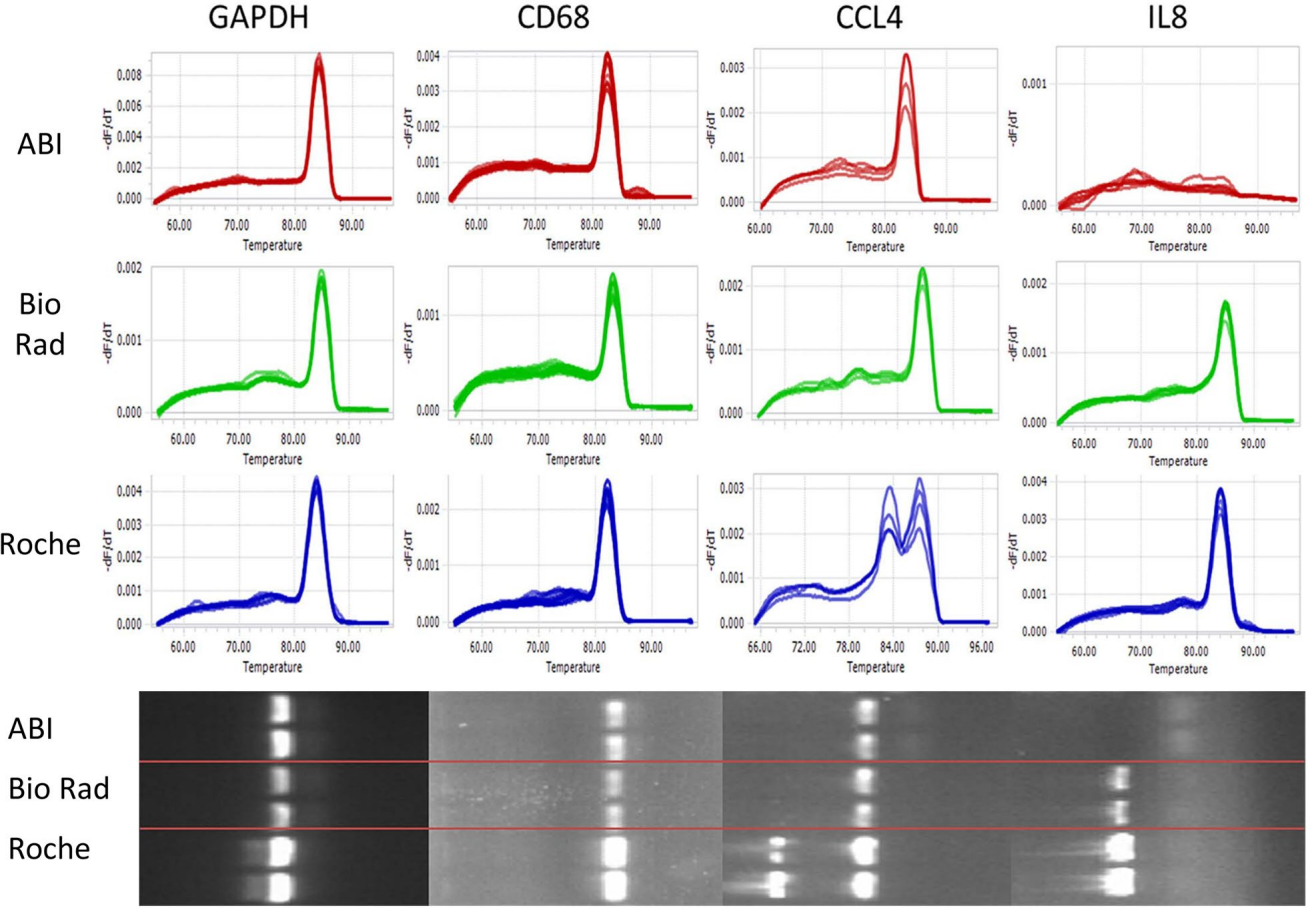
Not all PCR mixes result in optimal specificity of cDNA amplification reactions. The performance of three commercial SYBR green PCR mixes was compared by amplifying cDNA with 70 primer-based assays targeting different mRNA transcripts. A single melting curve peak indicates specificity of the amplification. The figure shows examples of melting curves and corresponding gel blots for several primer sets from Table 3, in situations where all three mixes gave optimal results and where one or more mixes resulted in a suboptimal amplification reaction

Melting profiles represent a suitable means to distinguish amplified products from primer dimer and other nonspecific amplification artifacts [8, 18]. In terms of cDNA templates, 79 immune-related transcripts were targeted by specific primer pairs in PCR reactions containing ABI, Bio Rad or Roche PCR Mix on a Light Cycler 96 PCR device. Of these, nine primer pairs showed low performance due to either the absence of amplification product or nonspecific amplification with any of the three different mixes. These were left out of further analysis. The remaining 70 transcripts were classified into four categories according to the melting profiles obtained after PCR with the three different master mixes (Table 3). Sixty-six primer sets (94.3 %) generated a single sharp melting peak with all three SYBR green PCR mixes in case of adherence to the suggested PCR protocol in the vendors’ guidelines (Table 3, category 1a). In case of using Roche mix in combination with a general lab PCR protocol (Table 2), 13 primer pairs (18.6 %) led to suboptimal melting peak after the PCR indicating generation of a specific PCR products (Table 3, category 1b). The primer pair targeting CCL4 showed sharp and specific melting curves only with the ABI and Bio Rad master mix (category 2), while CCL18 showed a single and smooth melting peak only with the Roche mix (category 4). Two primers pairs (those targeting IL8 and IL4; category 3) demonstrated one sharp melting peak with Bio Rad and Roche but negative amplification with ABI mix. Representative melting profiles and gel plots for the categories are shown in Fig. 1.

**Table 3:** Categories classified by amplification specificity

Cat	ABI	Bio-Rad	Roche	Transcripts	Number
1a	Y	Y	Y	GAPDH, CD23, CD68, TLR9, Arg1, PDL1, CXCR4, COX2, B-actin, CXCR1, CCL2, CCL3, CD115, CD117, CD11b, CD163, CD14, CD66b, CD86, HLA-DR, IL10, HO-1, IL1b, IL6, S100A9, STAT4, STAT6, STAT3, TGFB1, TNFa, CCL5, CCL7, V-JUN, CSF3R-2, CD13-2, CCR5, CD31, CD44, CD54, CD64, CD16a, CD205, NFkB, S100A8, CCR2, CD62L, MSR1, CCL24, CD15, CD209, CLEC4C, FLT3, IFNγ	66
1b	Y	Y	Y/N^a^	IL-1RN, IL-18, CEBPB, v-FOS, Egr1, Egr2, CD54, CD200R, CD40, CD1c, TLR2, CD43, CCR7	
2	Y	Y	N	CCL4	1
3	Neg	Y	Y	IL8, IL4	2
4	Neg	N	Y	CCL18	1

*Y* a single smooth sharp peak; *N* more than two or unsmooth peaks; *Neg* no amplification

^a^With Roche mix, the primers generated specific PCR amplicons in the melting curve analysis, only when the suggested PCR protocol from the vendor’s guideline (Table 2) was used. With a general lab PCR protocol (Table 2) suboptimal melting curves were observed indicating additional aspecific PCR products

**Fig. 2:**
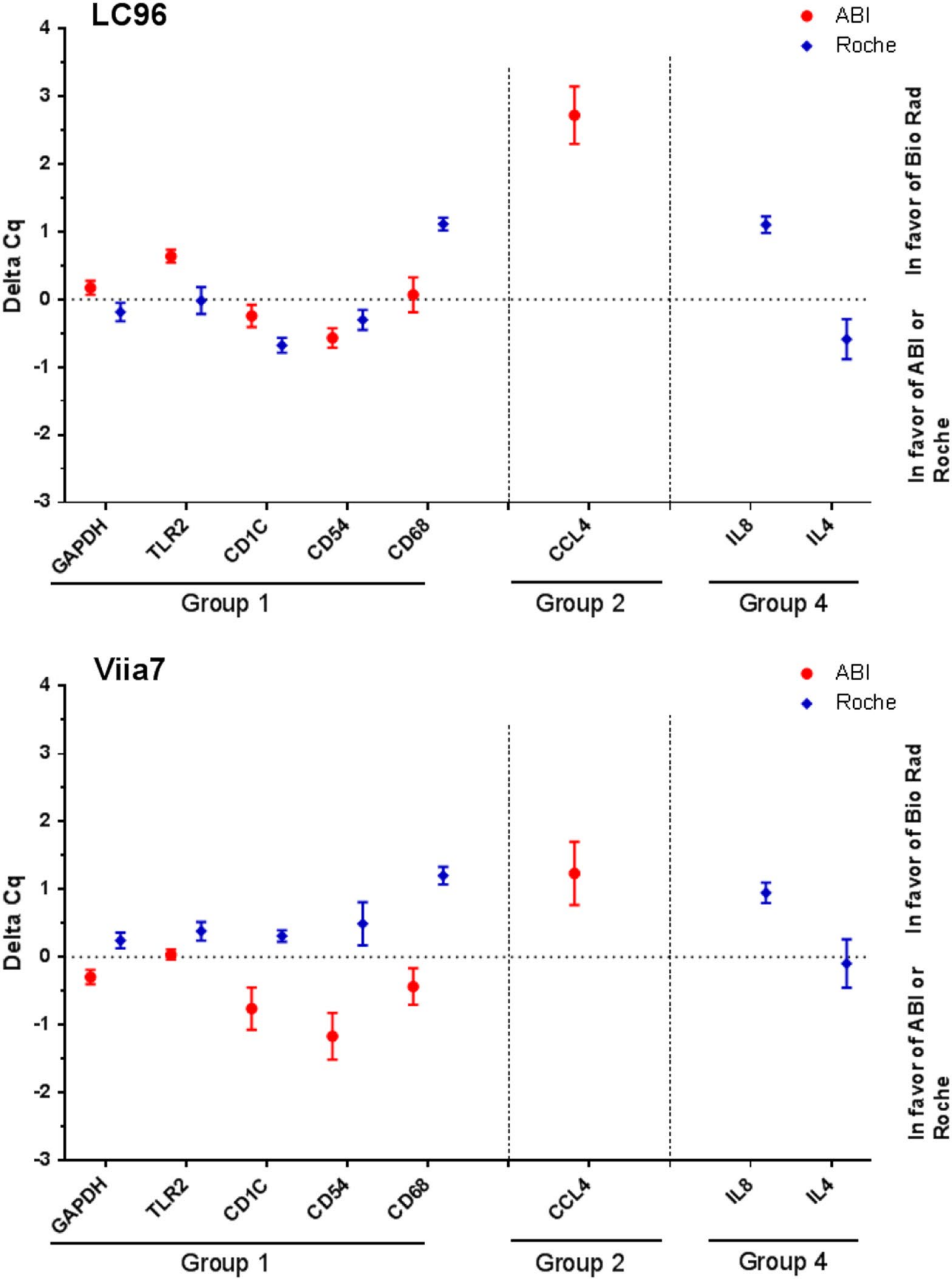
Delta-Cq values between different PCR mixes after cDNA amplification with only those primer pair/PCR mix combinations which led to one specific melting peak. Results for BioRad PCR mix represent the reference (set to zero; *black dotted line*). *Red* and *blue flags* represent results obtained with mixes from ABI and Roche, respectively

The Cq value is another relevant outcome parameter in quantitative PCR. The difference in Cq value between different PCR mixes was only calculated for the primer sets that gave a specific PCR product with at least two mixes (Fig. 2). Delta Cq between PCR mixes varied according to the transcript analyzed and the PCR machine that was used. GAPDH, TLR2, and CD1c showed lower Cq values by Roche mix on a LC96, while lower Cq values were obtained by ABI mix on a ViiA7. Two primer pairs (CD54 and CD68) generated lower Cq values by ABI mix compared with others, which was most prominently observed when using the Viia7 machine. The primer pair of CCL4 produced higher Cq values by ABI mix than the Bio Rad mix on both instruments. Transcript targeting IL8 demonstrated higher Cq values by Roche mix than by Bio Rad mix, whereas IL4 showed lower Cq values by Roche mix on two machines (Fig. 2).

Amplification signals in the no template control (NTC) sample are indicative for primer dimer formation or contamination problems [19]. The Bio Rad and Roche mix occasionally showed positive signals with high Cq values (Cq >40) in NTC, while the ABI mix exhibited negative amplification (Cq >45) in most cases (Additional file 1: Figure S1). On minus-reverse-transcriptase controls the ABI mix generated negative amplification (Cq > 40) more frequently than the other mixes (Additional file 1: Figure S1).

### Amplification of genomic DNA

**Fig. 3:**
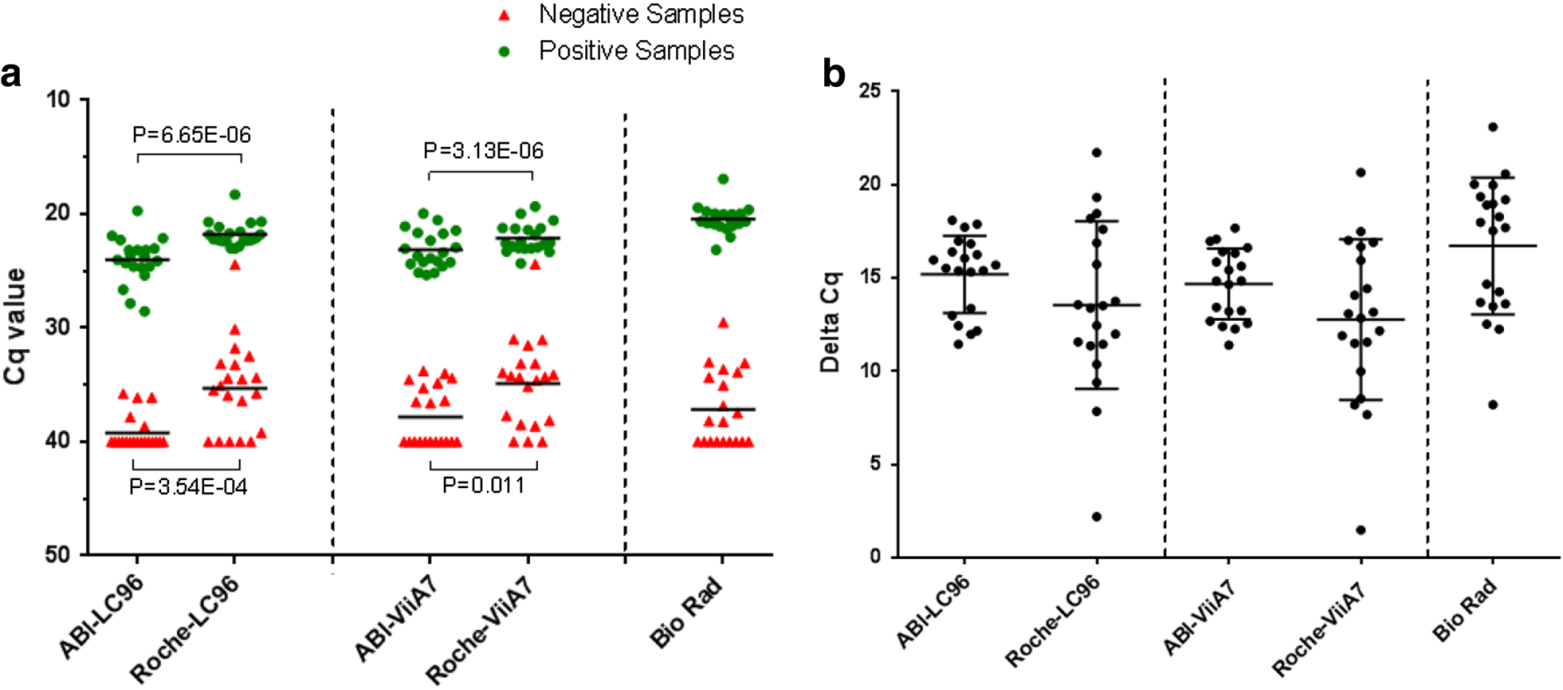
Cq difference between positive and negative genomic DNAs obtained with different PCR mixes and machines. **a** Cq values of 20 primer-selective PCR SNP assays for gDNA samples that should be positive (*green dots*) or negative (*red squares*). **b** Individual delta-Cq values for 20 primer-selective PCR SNP assays between positive and negative gDNA samples for ABI and Roche PCR mixes on two different PCR machines. The *flags* indicate means ± SD

Twenty primer-selective PCR SNP assays on genomic DNA were conducted on two different PCR devices. An optimal annealing temperature of 61 °C was employed, as tested in a temperature gradient. Absolute Cq values for DNA samples that should be positive or negative for the targeted SNPs are shown in Fig. 3a. The mean ΔCq for the 20 assays between positive and negative genomic DNAs was higher with the ABI mix than with the Roche mix (Fig. 3b), but this difference was not significant. However, of all mixes tested, the use of ABI mix led to the smallest variation in ΔCq among the different PCR assays (Fig. 3b).

### Genotyping by HRM

**Fig. 4:**
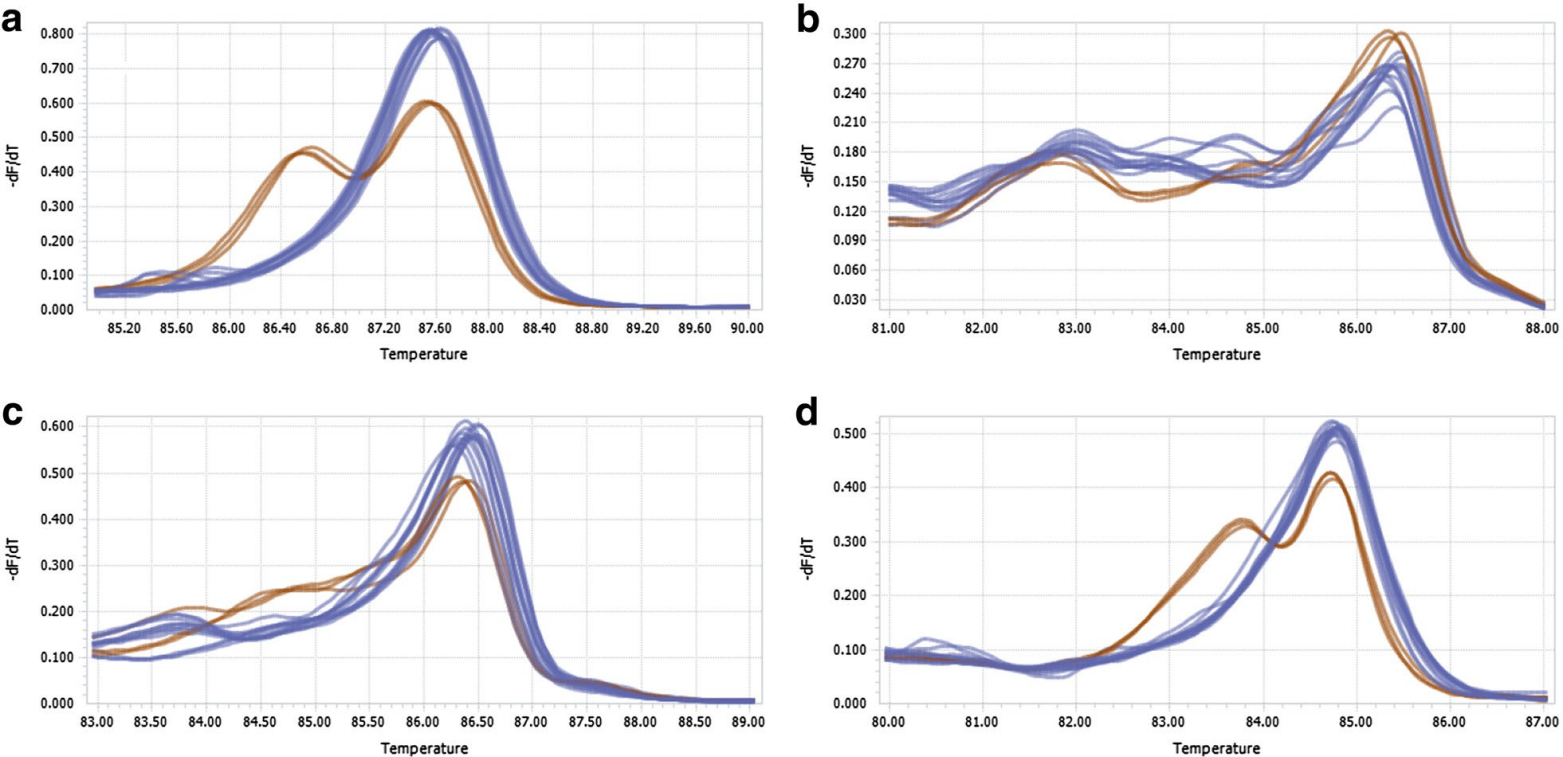
Effect of the type of high resolution melting (HRM) PCR mix on melting curve profiles for distinction of different genotypes. Three DNA samples heterozygous (GC) and 12 DNA samples homozygous (GG) at position rs2230199 were genotyped with HRM using either, **a** high resolution melting master (Roche), **b** qPCRBIO HRM Mix(PCR Biosystems), **c** SensiFast HRM Kit (Bioline), or **d** MeltDoctor HRM Master Mix (Applied Biosystems by Life Technologies). Genotypes were correctly classified with Roche and ABI HRM mixes

For high resolution melting analysis the fluorescent data were automatically normalized and derivative melting curve plots were generated (Fig. 4). Both the Roche (panel A) and ABI HRM mix (panel D) were able to distinguish the three heterozygous samples (GC, orange lines) from the 12 homozygous samples (GG, blue lines). The melt curves from Roche HRM mix were more tightly grouped and easier to separate into clear clusters than ABI HRM mix. With the Bioline HRM mix (panel C) it was also possible to correctly classify the DNA samples according to the right genotype, but the curves were rather unsmooth and tangled. With the PCR Biosystems mix (panel B) none of the three heterozygous DNA samples were correctly classified.

## Discussion

Real time PCR technology has been widely accepted because of its high specificity, sensitivity and reproducibility. Selection of appropriate kits is relevant for obtaining reliable results. Here we presented the performance of various SYBR green PCR mixes and HRM mixes. We wanted to test the robustness of different commercial SYBR green PCR mixes with respect to specificity and sensitivity of the PCR assay.

Sieber and colleagues have shown substantial performance discrepancies among commercial cDNA synthesis kits and qPCR kits in three species (mouse, rat, human) [20]; the current study mainly focused on the RT-qPCR process, thereby including specificity of the PCR assays as an essential outcome parameter. Melting curve analysis following PCR amplification can identify the presence of nonspecific amplicons [8, 18]. For a subset of primer pairs the melting profile exhibited differences between PCR kits when using one distinct PCR program. However, the poor melting profile markedly improved once the prescribed protocol were strictly followed. This improved amplification may result from the increased extension temperature of the Roche PCR program. Overall, 66 out of 70 transcripts showed a single smooth sharp peak by all commercial PCR kits (Table 3). The transcript targeting CCL4 demonstrated two melting peaks by Roche mix and the PCR products showed two bands in the gel plot. The primer pairs of IL-8 and IL-4 exhibited negative amplification and absence of PCR products by ABI mix. This discrepancy between transcripts may result from differences in magnesium chloride concentrations between PCR mixes.

When measuring the mRNA expression levels, the PCR amplification efficiency is particularly important [21]. The primer sets (GAPDH, TLR2, CD1c, CD54, CD68, CCL4, IL8 and IL4) used for Cq comparison among mixes displayed an acceptable amplification efficiency (Table 3). Two transcripts (CD54, CD68) showed lower Cq values by ABI mix compared to the other mixes on both machines, with even larger disparity on the ViiA7. Interestingly, the CCL4 or IL8 exhibited smaller Cq values by Bio Rad mix than ABI mix or Roche mix, respectively. The inconsistencies in amplification efficiency, especially in categories 2–4, may be due to differences between reagents such as salt concentration and acidity of the solution. Lu showed differences for four genes between ABI and Roche (LC480) PCR systems and also critical effects of magnesium concentration [22]. In the current study, we also showed that the ΔCq values between Roche and Bio Rad mix were slightly smaller on the LC96 than on the ViiA7, and similarly, ΔCq values for ABI and Bio Rad mixes were lower on the ViiA7. Therefore, the PCR kit and equipment from the same company are compatible with each other.

DNA chimerism analysis is a useful means to monitor the patient after transplantation, and the PCR assays used for this require high specificity [17, 23]. We found that different SYBR green mixes had a different capacity to distinguish positive and negative DNA samples. Although the mean ΔCq between positive and negative DNA samples were not significantly different between PCR mixes, the variation in ΔCq between assays with the ABI mix was smaller than with the Roche and Bio-Rad mixes. This was seen on two different PCR machines. Therefore, we conclude that the ABI PCR mix gives the highest consistency among 20 primer-selective SNP assays on DNA samples.

HRM is a powerful and flexible technique that can be used for genotyping and mutation scanning. The saturating dsDNA-binding dye is one of the important factors for successful HRM analysis. Both Roche and ABI mix could correctly identify the genotype of DNA samples under the identical PCR program conditions (Fig. 4). In contrast, the other two HRM mixes generated tangled and unsmooth melting curves, probably because of the quality of PCR amplicon. Our results showed that the source of HRM master mix is a major determinant of successful HRM analysis.

## Conclusions

Our data show that three commercial PCR mixes exhibit significant differences with respect to sensitivity of the PCR assay when applying a large panel of primer sets for mRNA transcript quantitation. The consequences of the current findings are that the use of ABI mix has a preference because of higher robustness: this mix more often led to lower Cq values and a specific PCR reaction, also in case of deviating PCR protocols, compared to other mixes. With primer-selective amplification of genotype variants in genomic DNA samples, ABI PCR mix led to lower background level for negative samples and smaller variation among different assays between positive and negative genomic DNA samples. Overall, the source of the PCR mix had a greater influence on the results than the PCR device used. Finally, with HRM analysis of genomic DNA samples, PCR mixes from Roche and ABI produced the most distinctive melting profiles for correct genotype classification. The present results show that the type of master mix used in nucleic acid amplification reactions determines specificity of the assay and PCR yields.

## Additional file

10.1186/s13104-016-2093-4 Absolute Cq values by three master mixes on no template controls (NTC) and minus reverse transcriptase (−RT) controls.

## Abbreviations

HRM: high resolution melting
SNP: single nucleotide polymorphism
PCR: polymerase chain reaction
qPCR: quantitative PCR
DNA: deoxyribonucleic acid
Ds DNA: double-stranded DNA
cDNA: complementary DNA
gDNA: genomic DNA
ABI: applied biosystems
LC 96: light cycler 96
NTC: no template control
−RT control: minus reverse transcriptase control
