# Supplementary figures and images for: The source of SYBR green master mix determines outcome of nucleic acid amplification reactions
Source: BMC Res Notes. 2016 Jun 4;9:292. doi: 10.1186/s13104-016-2093-4 (PMC4893258; doi:10.1186/s13104-016-2093-4)

## Slide 1
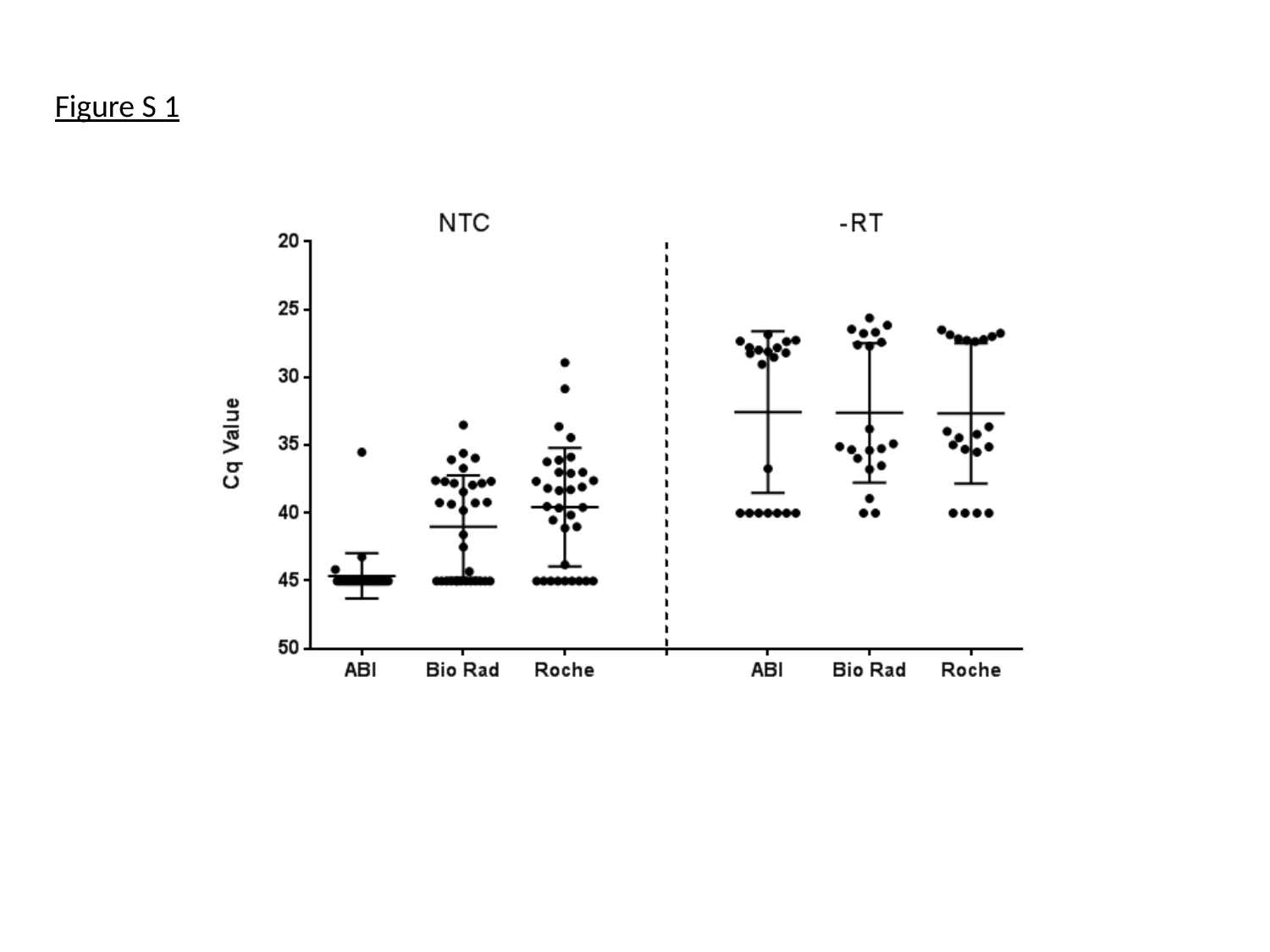

Figure S 1

Supplement: Supplementary file 1 — 10.1186/s13104-016-2093-4 Absolute Cq values by three master mixes on no template controls (NTC) and minus reverse transcriptase (−RT) controls. [file 13104_2016_2093_MOESM1_ESM.pptx]
